# Doctoral researchers’ mental health and PhD training satisfaction during the German COVID-19 lockdown: results from an international research sample

**DOI:** 10.1038/s41598-022-26601-4

**Published:** 2022-12-22

**Authors:** Sandra Naumann, Magdalena Matyjek, Katharina Bögl, Isabel Dziobek

**Affiliations:** 1 Berlin School of Mind and Brain, Humboldt-Universität zu Berlin, Berlin, Germany; 2 Department of Psychology, Institute of Life Sciences, Humboldt-Universität zu Berlin, Berlin, Germany

**Keywords:** Health policy, Risk factors, Population screening

## Abstract

Academia has been facing a mental health crisis particularly affecting early career researchers (ECRs). Moreover, the COVID-19 pandemic posed an unprecedented burden on the mental health of many individuals. Therefore, we cross-sectionally investigated how doctoral researchers (N = 222) evaluate their mental health status and satisfaction with their PhD training before and during the pandemic. As compared to self-reported, retrospective evaluations about the pre-pandemic state, we found decreased satisfaction with PhD training and overall well-being. The whole sample exhibited high levels of personal and work-related burnout, a fifth indicated clinically meaningful levels of depressive symptoms and almost 25% experienced severe loneliness. When exploring predictors of depression, anxiety, and burnout, we identified low satisfaction with PhD training as the most prominent predictor for poor mental health, suggesting a link between the doctoral work and their mental health status. Females vs. males and doctoral researchers in individual doctorate vs. structured PhD programs reported higher symptoms of burnout. Our study replicates previous findings of poor mental health in doctoral researchers and indicates further decreases of mental wellbeing under the influence of the pandemic. Systematic adjustments in academia are required to improve the mental health of ECRs.

## Introduction

Although many academics indicate to love research and to experience fulfilment from various tasks which belong to their profession^1^, mounting evidence suggests that working in academia might contribute to mental health problems^2,3^. Early career researchers (ECRs), i.e., doctoral and early postdoctoral researchers, have been found to be at risk for mental disorders with prevalence rates up to 24% for depression and 17% for anxiety^4^. These levels are up to three times higher as compared to the prevalences in the general population^5,6^. Consequently, it has been suggested that academia faces a “mental health crisis”^2^, sparking a discussion of its causes and possible remedies.

Among the main stressors for ECRs are the unpredictable length of the PhD training, financial instability, and high competitiveness for subsequent academic jobs^4^. Further, the prevailing ‘publish or perish’ culture pressures academics to put quantity over quality in their scientific work^7^, increasing stress and work dissatisfaction. Poorer work-life balance and higher conflict between work responsibilities are also linked to higher burnout rates in ECRs^8^. Other, not directly work-related, factors such as feelings of loneliness^5^ also have been shown to negatively affect their mental health.

With the onset of the COVID-19 pandemic, mental health worsened in the general population with elevated levels of depression, anxiety, and feelings of loneliness^9,10^. First studies indicate that ECRs' mental health was also negatively affected^10^. Pandemic-related changes imposed additional major stressors on ECRs^11^: Empirical work, which involved interactions with participants, had to be terminated for longer periods of time causing unforeseeable delays in PhD projects^12^. Social distancing forced researchers to stay at home, isolated from their support networks of colleagues. This might have been especially challenging for first year doctoral researchers and international doctoral researchers, who were disconnected from previous social networks and had limited chances to establish new ones after their relocation. For ECRs with children and family care duties, personal responsibilities increased as kindergarten and eldercare facilities closed, which had a disproportionate impact on female scientists^13^.

Taken together, the pandemic accumulated many additional problems on ECRs^11^ who were shown to be already at high risk for developing mental health problems due to the pressure and working conditions in the academic system^2–4^. So far, specific research on the impact of the COVID-19 pandemic on ECRs’ mental health is scarce, however, and mostly limited to anecdotal evidence^14^. Therefore, we assessed the mental health status of a defined group of ECRs, particularly doctoral researchers, under the consideration of pandemic influences. Data were collected in Berlin, a central German research hub with numerous international research institutions. Given the international science context, we assumed that our results would be informative beyond national borders.

Firstly, we sought to assess the current mental health of doctoral researchers including existing clinical diagnoses of mental disorders, acute symptoms of common mental problems (i.e., depression, anxiety, burnout, loneliness), and support structures (e.g., strength of social network, individual coping strategies). In accordance with the literature^3,4^, we expected to find elevated rates for depression and anxiety symptoms in our sample. Secondly, we aimed to examine the effects of the COVID-19 pandemic on doctoral researchers’ mental health and satisfaction with the PhD training. During data collection (January and February 2021), the pandemic status had been officially declared for almost a year in Germany (starting in March 2020). By then, two waves of infection had been observed and a strict lockdown had been imposed on the public for almost two months, with restrictions on public life still effective at the time of data collection. We assumed that, given the additional stressors implied on research by the pandemic, satisfaction would decrease for various PhD-related aspects when comparing ratings of pre-pandemic times with the current, in-pandemic, situation. Further, we expected that many aspects related to the PhD training would contribute more to mental health problems in the pandemic, compared to how much they did before its onset. Thirdly, in an exploratory analysis, we investigated potential predictors of doctoral researchers’ depression, anxiety, and burnout symptoms. Given the exploratory nature of this procedure, hypotheses were more tentative: We assumed that higher PhD training satisfaction and integration into a structured graduate program would be associated with lower symptom scores.

## Results

### Mental health status

#### Standardized questionnaires concerning mental health

Table 1 includes an overview of the results for BSI, CBI, and De Jong Gierveld Loneliness Scale scores. Regarding the BSI, 21% of the sample exceeded the threshold for depression and 4% for anxiety. Means of the CBI subscales for personal burnout and work-related burnout were 49 and 47, the mean score in the CBI total scale was 48 (including only the personal and work-related burnout subscales). Both subscale scores were substantially higher than the means in the validation sample^15^. Regarding loneliness, 24% of the sample indicated to be severely or very severely lonely (scores 9 or higher).

**Table 1:** Means (M) and standard deviations (SD) for the standardized mental health measures divided by gender. Note: Participants who indicated “prefer not to answer” (N = 5) were not included in the table.

Brief Symptom Inventory									
Gender	*M*	*SD*	≥ 1 *SD*	*M*	*SD*	≥ 1 *SD*	*M*	*SD*	≥ 1 *SD*
	**Depression**			**Anxiety**			**Phobic anxiety**		
All	55.7	3.8	N = 47	52.1	4.7	N = 8	59.0	4.5	N = 124
Woman	55.1	3.7	N = 30	51.2	4.6	N = 1	59.0	4.9	N = 84
Man	56.7	3.8	N = 14	53.7	4.5	N = 7	59.0	3.4	N = 35
Other	58.8	2.9	N = 2	54.5	2.9	N = 0	61.0	7.1	N = 2
	**Obsessive compulsive**			**Hostility**			**Psychoticism**		
All	49.8	4.4	N = 0	52.3	5.2	N = 18	57.4	4.0	N = 42
Woman	49.4	4.7	N = 0	51.3	5.0	N = 4	57.6	4.4	N = 27
Man	50.4	3.8	N = 0	54.5	5.0	N = 14	57.0	3.2	N = 14
Other	51.3	2.9	N = 0	52.5	5.5	N = 0	57.8	2.5	N = 0
	**Paranoia**			**Sensitivity**			**Somatisation**		
All	52.6	3.6	N = 6	54.3	5.2	N = 45	51.8	3.7	N = 12
Woman	52.5	3.6	N = 3	53.1	5.0	N = 23	51.1	3.4	N = 1
Man	52.8	3.6	N = 3	56.3	5.1	N = 20	53.2	4.0	N = 11
Other	53.5	5.2	N = 0	58.3	3.7	N = 1	52.3	4.0	N = 0

Copenhagen Burnout Inventory									
Gender	*M*	*SD*	*M*	*SD*	*M*	*SD*			
	Personal		Work		Total				
All	49.1	21.8	47.1	22.0	48.0	20.4			
Woman	52.1	21.5	49.1	21.8	50.5	20.1			
Man	43.2	21.2	42.7	21.8	42.9	20.1			
Other	56.3	24.9	63.4	20.5	60.1	21.8			

De Jong Gierveld Loneliness Scale									
Gender	*M*	*SD*	*M*	*SD*	*M*	*SD*	≥ 9		
	Social		Emotional		Total				
All	2.1	1.8	3.7	1.7	5.8	3.1	N = 54		
Woman	2.0	1.8	3.7	1.7	5.6	3.0	N = 34		
Man	2.6	1.9	3.7	1.8	6.3	3.3	N = 19		
Other	2.3	1.5	4.3	1.7	6.5	1.3	N = 0		

#### Mental disorder manifestation, mental health problems, and expectations related to the PhD training

**Figure 1:**
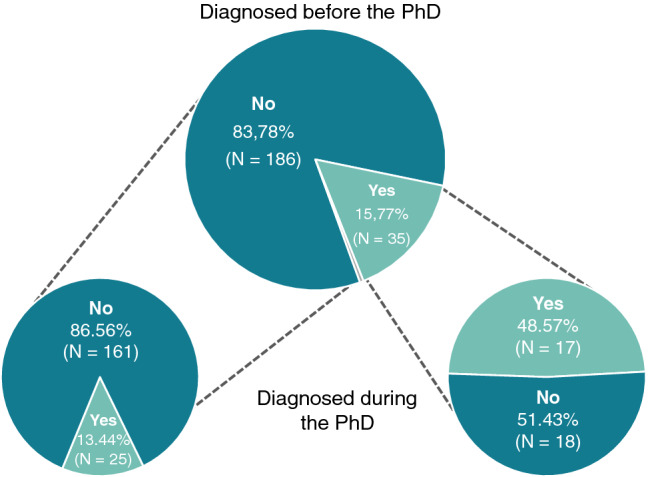
Diagnosis of a mental disorder. The top chart shows proportions of participants diagnosed with a mental disorder or not prior to the beginning of the PhD training (1 person preferred not to answer this question; in grey). Separately for those who answered yes and no, bottom charts show proportions of participants further diagnosed and not diagnosed with a mental disorder during their PhD training. Please note: One person preferred not to answer.

Participants were asked whether they had been diagnosed with a mental disorder before or after the start of their PhD training. As depicted in Fig. 1, 16% of the respondents indicated that they received a mental disorder diagnosis before they started their PhD training. From this group, 49% stated that they were diagnosed with an additional mental disorder after the start of their doctorate. A majority of 84% indicated that they were not diagnosed with a mental disorder before. From this group, 13% indicated that they got diagnosed with a mental disorder after they started their PhD training. In total, 27% of doctoral researchers reported at least one clinically diagnosed mental disorder before or after the start of their PhD training.

**Figure 2:**
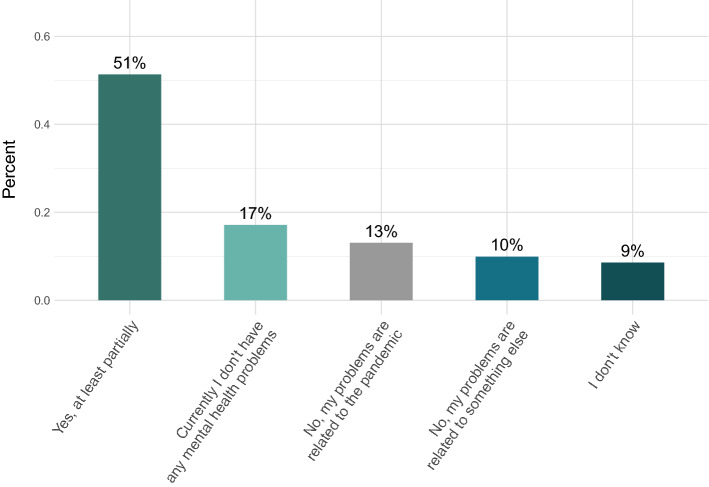
Mental health problems in relation to the PhD training. Participants were asked whether they think that their current mental health problems are related to their PhD training.

**Figure 3:**
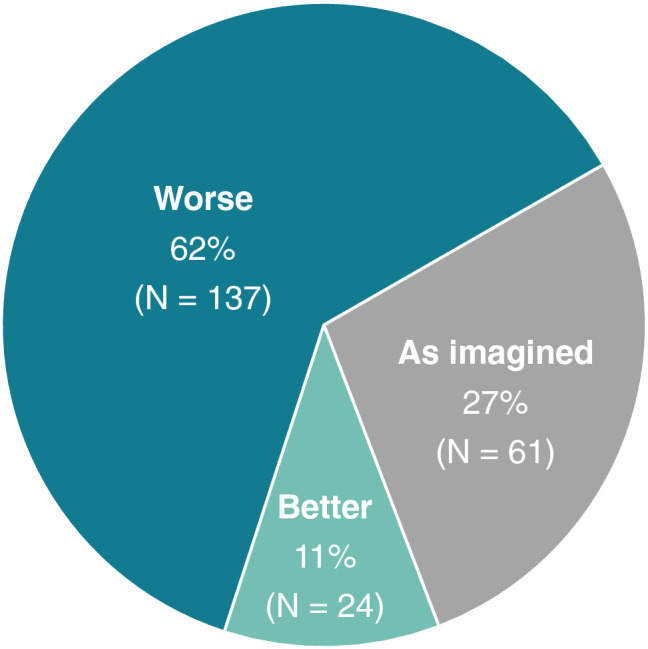
Expectations of the PhD training. Participants were asked whether their PhD training is how they had imagined it, better, or worse. Answers including “slightly” and “much” for both “worse” and “better” were collapsed.

As shown in Fig. 2, the majority of doctoral researchers stated that their current mental health problems were at least partially related to their PhD training (51%), whereas only 17% reported to have no mental health problems. 62% of the responders indicated that their PhD training is a little or much worse than they imagined before they started it (see Fig. 3).

#### Support structures

Only 16% of respondents expressed no interest in receiving psychological support. In contrast, 40% of doctoral researchers endorsed the answer “Yes, I would like it (or I am receiving some)” and 39% “Yes, I would like it, but I don’t need it now”. The forms of support rated as of most interest were psychotherapy in person (62%), psychological counselling (52%), and psychotherapy online (40%). Further, respondents stated that the coping strategies they use the most comprise social engagements (indicated by 85%) and recreational activities (78%; multiple answers were possible). To further delineate the role of social engagements, we asked participants to rate the strength of their overall social network (*M* = 73; *SD* = 23) and the strength of their social network in Berlin (*M* = 53; *SD* = 31). Further information on the forms of support as well as coping strategies are provided in the supplementary material (see the HTML file on the OSF page).

### Changes in PhD training satisfaction and mental health during the pandemic

**Figure 4:**
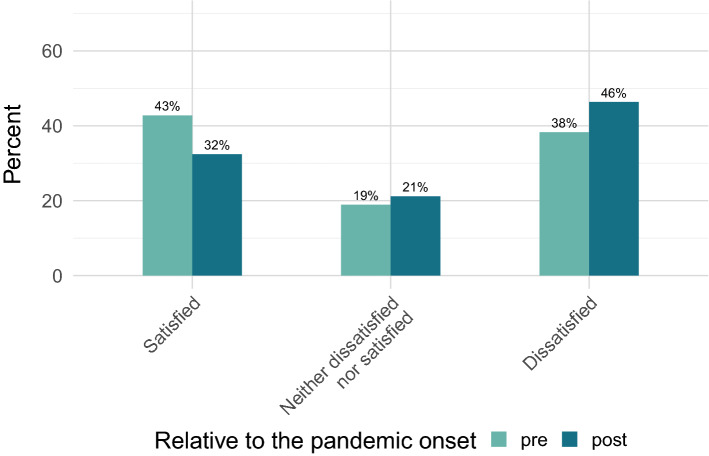
Overall satisfaction with the PhD training before and during the pandemic. Answers including “very” and “somewhat” for both “satisfied” and “dissatisfied” were collapsed.

**Figure 5:**
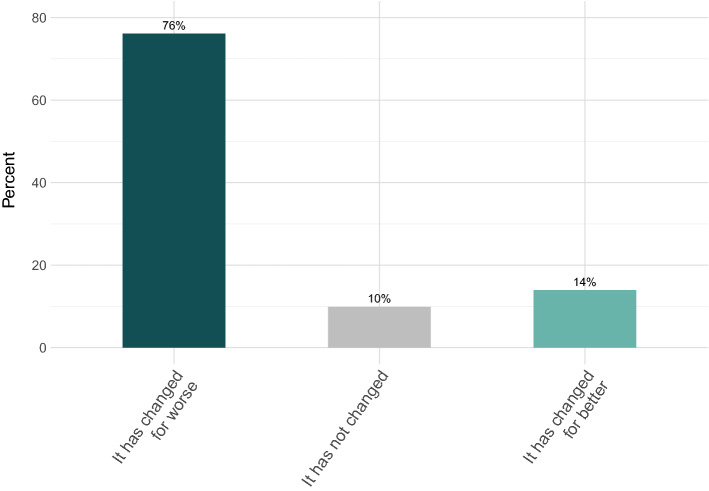
Mental wellbeing in the pandemic relative to pre-pandemic. Answers including “significantly” and “slightly” for both “worse” and “better” were collapsed.

As shown in Fig. 4, 43% of the respondents indicated that they were satisfied with their PhD training before the pandemic started, whereas only 32% stated that they were satisfied with it currently, in pandemic times. Evaluating the pre-pandemic situation, 38% of the sample indicated that they were dissatisfied, which increased to 46% after the start of the pandemic. Regarding the perceived change in wellbeing before and in the pandemic (see Fig. 5), 76% indicated that their mental wellbeing worsened during the pandemic.

**Figure 6:**
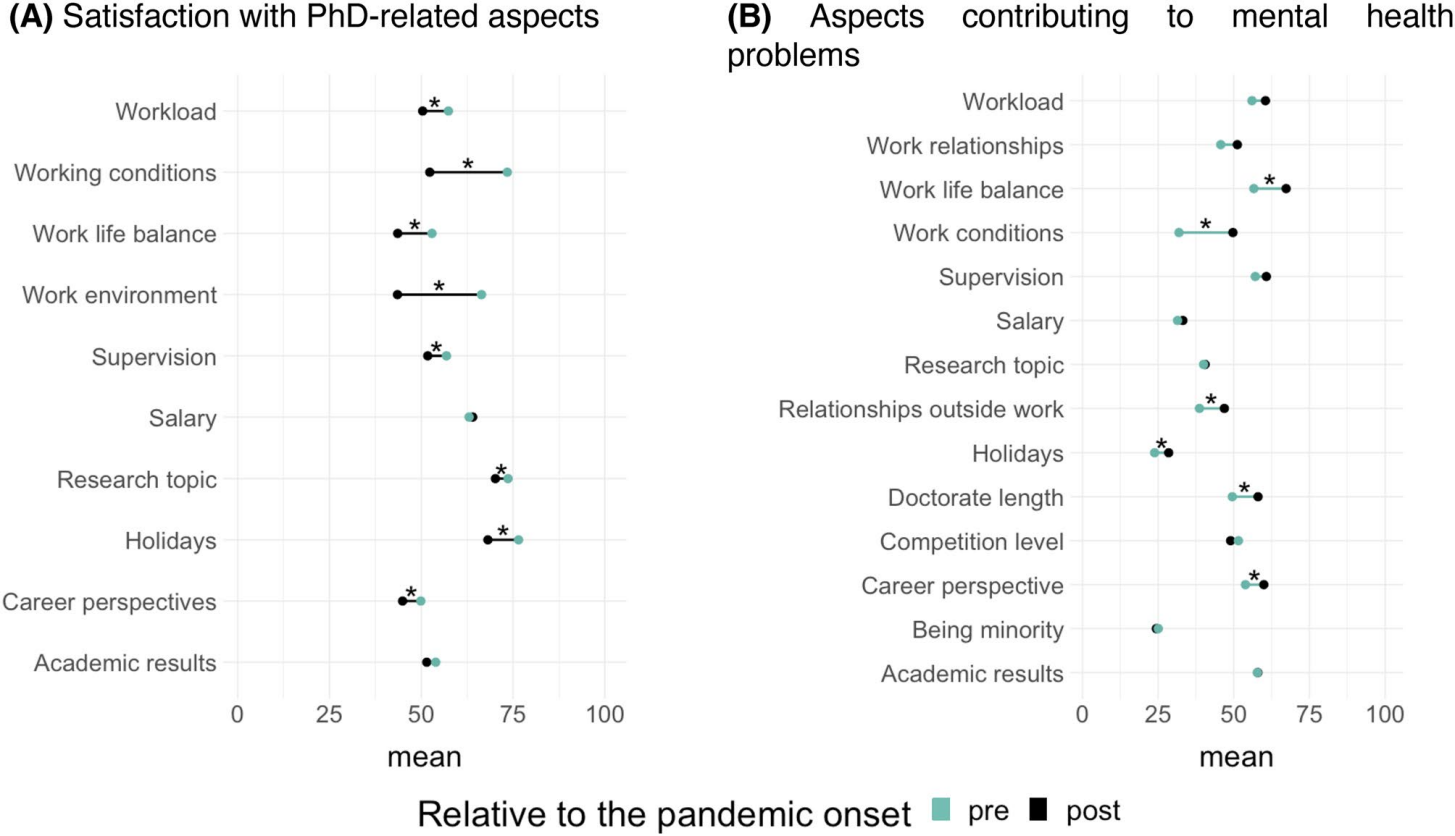
Average ratings of satisfaction with PhD-related aspects and of aspects contributing to mental health problems pre- and post-pandemic onset. The asterisks mark items for which t-tests reached statistical significance (*p* < 0.05, uncorrected).

As shown in Fig. 6A, respondents evaluated their satisfaction with PhD-related aspects generally lower after than before the onset of the pandemic. Pre-pandemic, doctoral researchers were descriptively most satisfied with their research topic, working conditions, and holidays (all items rated at around 75%). We found the lowest satisfaction ratings for career perspectives, work-life balance, and academic results (the lowest at 50%). In pandemic times, research topic, salary, and holidays were rated highest (highest averaged rating at 68%); work-life balance, career perspectives, and work environment were rated the lowest (the lowest at 43%). We found significant decreases in the pandemic in self-rated satisfaction from workload, work environment, working conditions, work-life balance, supervision, research topic, career perspectives, and holidays, (all *ps* < 0.01). The satisfaction from academic results and salary did not change significantly during the pandemic.

Participants also rated how these aforementioned aspects contributed to their mental health problems before and after the beginning of the pandemic (see Fig. 6B). Descriptively, the aspects that contributed most to mental health issues pre-pandemic were academic results, supervision, work-life balance, and workload. In pandemic times, the major contributor was work-life balance, followed by workload, supervision, and career perspectives. We observed significant increases for the ratings of career perspectives (*p* = 0.002), work-life balance (*p* < 0.001), relationships outside of work (*p* = 0.003), and holidays (*p* = 0.043), indicating that in the pandemic these factors played a larger negative role for the mental health of doctoral researchers. Other aspects did not change during the pandemic (See OSF page for exact t-values).

### Predictive value of PhD training variables for mental health problems

**Figure 7:**
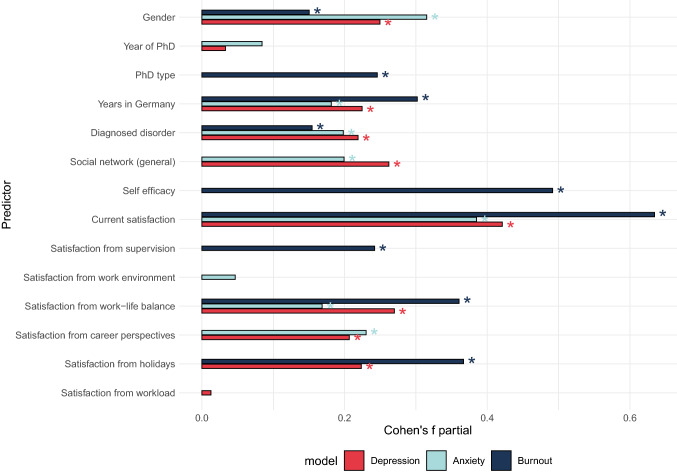
Effect sizes (Cohen’s partial *f*) in the exploratory models of depression, anxiety, and burnout. The asterisks mark predictors for which the main effects reached statistical significance (*p* < 0.05).

Figure 7 shows the effect sizes (Cohen’s partial *f*) for the terms selected in the stepwise selection process for models predicting the depression, anxiety, and (work-related) burnout scores. Table 2 summarizes the models’ statistics and directions of the effects. Gender was significant in all models: While males tended to show higher values for anxiety and depression, being female was predictive of higher burnout scores. Years into PhD training was not significantly related to any outcome variable. PhD type (individual doctorate vs. structured program) was only predictive of burnout scores, with higher levels of burnout for the PhD researchers pursuing an individual doctorate compared to being in a structured program. Years spent in Germany, although showing significant main effects in all models, did not survive corrections for multiple comparisons. The diagnosis of a mental disorder (acquired before or during the PhD) was consistently predictive of all three mental health problems (i.e., higher scores of depression, anxiety, and burnout). Lower scores of depression and anxiety were predicted by strength of the overall social network, while higher self-efficacy was linked to lower burnout scores. The overall current satisfaction with the PhD training predicted lower scores for mental health problems in all models. In terms of PhD-related aspects, those of the ratings which reached significance were all linked to lower scores of mental health problems. In burnout, these predictors were: supervision, work-life balance, and holidays; in anxiety: work-life balance and career perspectives; in depression: work-life balance, career perspectives, and holidays.

**Table 2:** Statistics and directions of the effects in the exploratory models for depression, anxiety, and burnout. Columns “direction” include information on whether increasing values of the predictor were linked to increasing ( >) or decreasing ( <) values of the dependent variables (questionnaire scores). Post-hoc comparisons with Holm correction were administered for “Years in Germany” in all models, but none of the contrasts survived the corrections.

		Depression model		Anxiety model		Burnout model	
		*F* value	Direction	*F* value	Direction	*F* value	Direction
Demographic and support structures	Gender	11.6***	M > F	18.6***	M > F	4.2*	F > M
	Year of PhD	0.2	<	1.3	<		
	PhD type					11.3***	Individual > Structured
	Years in Germany	4.7*	Native < 4 + < 0–3, no p_corr_ sig	3.1*	Native & 4 + < 0–3, no p_corr_ sig	8.5***	0–3 & native < 4 + , no p_corr_ sig
	Diagnosed disorder	8.9**	Yes > No	7.4**	Yes > No	4.5*	Yes > No
	Social network (general)	12.8***	<	7.4**	<		
	Self-efficacy					45.2***	<
Satisfaction with PhD aspects	Overall	33***	<	27.7***	<	75.3***	<
	Supervision					11**	<
	Work environment			0.4	>		
	Work-life balance	13.6***	<	5.3*	<	24.3***	<
	Career perspectives	7.9**	<	9.9**	<		
	Holidays	9.3**	<			25.2***	<
	Workload	0.03	>				
**Model statistics**							
*F* (df)		9.7 (11,186)		8.4 (10,187)		21.8 (10,187)	
*R*^*2*^_multiple_/*R*^*2*^_adjusted_		0.37/0.33		0.31/0.27		0.54/0.51	

*p < 0.05, **p < 0.01, ***p < 0.001.

Degrees of freedom for main effects’ F values:

Numerator df: 2 for “years in Germany” and 1 for all others.

Denominator df: depression model: 186, anxiety and burnout model: 187.

## Discussion

We examined different self-report measures capturing the mental health status of doctoral researchers of the wider Berlin area. Self-report ratings implied increased levels of depression, burnout, and loneliness, which further exacerbated under the pandemic influence. Critically, these problems were linked to the PhD training.

### Under the magnifying glass: doctoral researchers’ mental health status

Firstly, we investigated the mental health status of doctoral researchers regarding pre-existing clinical and subclinical manifestations. In line with previous research^2–4^, our participants displayed severe mental health problems. One quarter of the sample indicated to have at least one diagnosed mental disorder. Doctoral researchers with a pre-existing diagnosis more often developed an additional mental disorder during the years of their PhD training and the context of the COVID-19 pandemic. Thus our data suggests that negative mental health impacts seem to be magnified particularly for ECRs with pre-existing vulnerabilities. Comparable to previous findings, one fifth crossed the cut-off for clinically relevant depressive symptoms^16^. These numbers are higher than the depression prevalence in the general German population before (7.7%^6^) and during the pandemic (14.3%^17^). However, some studies which were conducted outside of Germany reported similarly elevated levels of depression in the general population within this time period^18^. Therefore, the increased depression levels in our sample might not just be attributable to the academic training but to the high mental burden caused by the pandemic itself. We also detected higher scores of burnout as compared to the general population^15^, paralleling recent findings presenting a three-fold increase of burnout in ECRs^11^. Alarmingly, the majority of the sample reporting mental health problems indicated that they were related to their PhD training. Moreover, most of our sample reported their PhD training experience to be worse than expected and that they would be interested in receiving psychological support.

Various PhD training-related aspects may underlie the reported mental health problems: Firstly, pursuing a doctorate constitutes a time of professional and personal growth^19^. ECRs need to master complex theoretical frameworks, develop expertise in research methodologies and make an original contribution to their field^20^. Additionally, establishing boundaries between work and other areas of life is challenging: Academics assume that pervasive commitment and profound concentration on the research subject are expected^21^. Increased workload may cause a neglect of physical health or personal relationships, leading to work-life imbalances^22^. Lastly, research facility structures which do not buffer the psychological and emotional costs of the PhD training may contribute to a deterioration of ECRs’ mental health^23^.

### Changes in satisfaction with the PhD training during the COVID-19 pandemic

Secondly, we aimed to unravel potential changes in satisfaction with the PhD training under the influence of the pandemic (which started about a year before this survey). We compared respondents' retrospective evaluation of their satisfaction before the pandemic with their current satisfaction. Almost all ratings significantly dropped during the pandemic with the largest decreases for working conditions and work environment. The German government introduced regulations to enforce social distancing to contain the COVID-19 virus, which also led to the closing of research facilities. Thus, a likely explanation for our results is that the disrupted or discontinued work on doctoral projects increased uncertainty and dissatisfaction with the PhD training^24^. Additionally, the workload and time spent on work might have increased significantly during the pandemic^25^, which corroborates the reported levels of increased work-related burnout in our survey.

There were no changes in satisfaction regarding doctoral researchers’ salaries during the pandemic. In contrast to the general German population, ECRs were not affected by Germany-wide short-time working measures (i.e., temporary reduction in normal working hours and thus reduction in salary). Further, at the time of the survey, the first pandemic-related extensions of PhD contracts and scholarships were granted. However, given that many empirical projects were on hold, the time until completion of the PhD training might prolong beyond the period of the granted extensions, which could increase the burden of financial insecurity in the long run. Although this hypothesis cannot be tested with the current data, it should be considered in future studies.

Likewise, we did not detect significant changes in satisfaction with academic results before and during the pandemic. It is possible that because some aspects of academic life (i.e., attending conferences) were limited, doctoral researchers had more resources to focus on output related to their PhD projects. In that vein, research indicated that the number of journal paper submissions increased during the pandemic^26^. Notably, our sample consisted of doctoral researchers which were at least three months into their PhD training in mostly empirical scientific fields. Given that they had already collected data for PhD-project related publications, they might have rated their academic results not to be significantly impacted by the pandemic.

### Predictive value of PhD training variables for mental health problems

Greater satisfaction with the PhD training in general, and with work-life balance in particular, were important resilience factors for all depression, anxiety, and burnout. This finding is in line with previous studies, which indicated that higher job satisfaction was associated with lower prevalence rates of depression and burnout in the general population^27^. Other studies highlighted that employees, who are satisfied with their work, can transfer positive feelings to non-work related contexts, resulting in a positive relationship between job and life satisfaction^28^, as well as psychological and social wellbeing^27^. Thus, maintaining ECRs’ satisfaction with their PhD training seems to be an essential mechanism to prevent mental health problems.

Further, our data showed that decreased satisfaction with PhD supervision is linked to burnout. Although we have no detailed information on the nature of the supervision quality, support from supervisors and other members of the research facilities have previously been reported to be critical to doctoral researchers’ persistence and scientific outcomes^29^. Research showed that the quantity and quality of meetings with supervisors can impact ECRs’ satisfaction^30^. Quality supervision can be characterized as involving precise and timely feedback, frequent meetings that include open discussion about roles and responsibilities, a supportive and collegial relationship, and encouragement to maintain the flow of work throughout the PhD training^31^. One driver of supervision dissatisfaction could arise from misaligned interests of ECRs and their supervisors: While the latter might be more inclined to tune their interest towards the scientific community as a whole, they might lose sight of ECR’s individual needs^32^.

In addition, we found that satisfaction with career perspectives is an important predictor of anxiety and burnout. It was recently argued that being optimistic about career prospects might help to decrease levels of depression and anxiety about the future^33^. However, long- and short-term academic career perspectives are uncertain, which may degrade academia to an “alternative career” path. Missing career outlooks, in or outside of academia, amplify the mental burden of ECRs, leading to the levels of anxiety and burnout^34^. This factor might be even more pronounced in countries of the Global South, where—compared to the Global North—levels of inequality and labour informality are higher and research funding is more limited^35^.

Within our analysis, the strength of doctoral researchers' social network emerged as an important resilience factor for depression and anxiety. Indeed, there is substantial evidence that individuals with richer networks of active social relationships tend to be more satisfied and happier with their lives^36^. However, due to the demands of their PhD training, ECRs often report declines in social interactions^37^. Lack of social support has also been found to correspond with lower wellbeing and a higher prevalence of mental illness in ECRs^38^. Similarly, in the current study ECRs who pursued an individual doctorate were more likely to suffer from burnout than their peers in structured graduate programs. Within a structured PhD program, doctoral researchers are integrated in a framework with peers from the start of the training, whereas individual doctorates require ERCs to build their own scientific community.

Another resilience factor identified in our data was self-efficacy, with higher levels being linked to lower burnout scores. Self-efficacy is an important motivational factor for identity development of ECRs^39^, especially when it comes to the confidence in successfully performing research tasks^40^. It has been found to be significantly correlated with interest in research and the production of scholarly publications^41^. Conversely, ECRs with low levels of self-efficacy may be more likely to engage in self-handicapping (e.g., procrastination) to avoid being perceived as incompetent^42^.

Finally, men in our sample showed higher levels of depression and anxiety symptoms than women. This finding is at odds with the literature, where women have been reported to suffer more often from mental health problems than men both in the general population^6^ and ECR samples^2^. Although this finding is difficult to interpret, previous research showed that men have lower participation rates in voluntary surveys^43^ and engage more rarely in help-seeking behavior^44^. Thus, it seems possible that the data reflect the effects of our convenience sample: Men may have participated in our survey because they experienced mental health problems, which resulted in increased clinical symptoms in this group. Further, although our male participants reported higher levels of depression and anxiety, female participants reported higher burnout scores. As shown in previous studies during the pandemic, women were more likely to engage in housework, care and family^13^, which relates to physical and psychological fatigue and exhaustion^15^, but less so to depression.

### Call to action

Our sample of doctoral researchers reported profound PhD training-related mental health problems, which worsened during the COVID-19 pandemic. Currently, mostly single researchers or initiatives founded by ECRs (e.g., German ECR initiatives like N^2^, Scholar Minds) devote their mission to reduce harmful work conditions and the stigmatization of mental health matters in academia. However, sustainable preventive and interventional solutions aiming to improve ECRs’ mental health should be prioritized and addressed on different systemic levels, such as academic institutions and political initiatives, to ensure a healthy and supportive work environment. Combining our findings with recent literature, we have identified several factors to improve conditions for ECRs. These factors are not exclusively tied to improving the situation regarding the COVID-19 pandemic but relate to more general aspects of ECRs’ mental health:

1. Institutional mental health support
  Firstly, psychological burden could be alleviated if the institutional culture of a research facility was more welcoming, inclusive, and understanding of ECRs’ backgrounds^20^. To this end, an open discourse about mental health problems is needed, which would also help to increase personal and public de-stigmatization of mental health problems still being present in academia^45^.
  Secondly, institution-based psychological counseling should be the standard at every research institution for prevention and interventional needs. Especially for international ECRs who are not familiar with the local health care system, institution-based counselling could be a first low-level opportunity to de-escalate arising mental health problems. Although some counselling opportunities are already in place, ECRs are often unaware of their existence (e.g., no advertisement of services; information difficult to access^46^). In addition, psychological counselling opportunities do not seem to be specifically tailored to doctoral researchers’ needs, but are rather similar to those designed for undergraduate students^47^. Thus, institutions would need to provide easily accessible PhD training-tailored psychological counselling.
2. Work-life balance
  Working overtime and disregarding holidays is still part of the academic culture^48^. With an average of more than 46 h of work per week^16^, doctoral researchers contribute to the normalization of this culture, likely neglecting their work-life balance. When employed part time, doctoral researchers have been shown to work even more over-time hours than postdoctoral researchers (difference of up to 7 h per week^48^). Irrespective of the type of working contract, the implicit rule to work overtime should be discouraged by employers and supervisors. Additionally, workshops on self- and time management provided by research institutions can strengthen ECRs’ ability to deal with the stressful demands of academia.
3. Quality of supervision
  Mentoring contracts between ECRs and supervisors could help to set expectations for both parties. This procedure is already in place at many research facilities, however, it is yet to become a common practice. Further, improving supervision quality should be incentivized by research facilities. Whereas ratings of teaching are already a part of applications for professorships, a similar quantification for supervision skills could maintain supervision quality over the course of the PhD training. Integrated evaluations by doctoral researchers could both incentivize senior academics to monitor their quality of supervision and offer tangible rewards for their efforts.
4. Providing career perspectives and transferable skills
  Even though staying in academia is still viewed as the most desirable career path for ECRs^49^, only a small percentage can continue to work in academia after the completion of their PhD training^50^. The creation of more permanent positions on a post-doctoral level is an important political challenge. In Germany, 98% of employees under 35 years of age are working on limited contracts in academia. Thanks to public attention on precarious working conditions in academia initiated by the German grass-root movement “#ichbinHanna” (https://ichbinhanna.wordpress.com), first political consequences that will lead to the creation of more tenure track positions, have been taken. Furthermore, opportunities to continue a career outside of science should be considered more strongly. Although many structured PhD programs have started to integrate educational content on alternative career paths, more room should be given for concrete opportunities to develop new skills as, for example, in the form of internships or role plays^51^. As skills acquired during the PhD training can successfully be transferred to careers outside of academia^34^, these real-life experiences might decrease anxiety regarding future job perspectives.
5. Growing social networks
  Research facilities play a major role in socializing ECRs^51^ which is why an important task constitutes the establishment of sustainable ECR networks in research facilities to grow sustainable social networks to discuss science matters and work-related challenges. Some graduate schools already provide induction days and buddy programs (e.g., connecting doctoral researchers across different PhD cohorts) to facilitate ECRs’ future academic career. In addition, regular check-ins with members of the research facility could help to identify and solve problems with settling into the research institution and its social network structure.
  Moreover, within- and between-institutional networks have the potential to help ECRs to shape their career paths. Some graduate schools are already providing alumni talks to connect ECRs with graduates from various career fields. This approach not only helps to expand the professional network, but also sharpens the view of which career options may be available after the doctorate and how to target these options early on.
6. Fostering self-efficacy

To maintain self-efficacy, the PhD training should entail tasks that are challenging, but achievable within the specific conditions of the doctoral project. Quite often, there is a mismatch in expectation between ECRs and research facilities^52^, likely arising from insufficient information at the admission stage regarding roles and responsibilities^53^. This parallels our finding that the majority of ECRs rated their PhD training experience markedly below their expectations. Thus, ECRs need to understand what explicit and implicit requirements are present toward the completion of their PhD training. One opportunity to enable clear expectation management is to offer an orientation day in graduate schools where potential doctoral candidates have the opportunity to receive information from administrative members, but also ECRs who are in different stages of their PhD training to provide testimonials.

### Limitations

Due to different potential biases in our sample, we acknowledge that our data might not be representative of the whole population of interest. As we might have mainly reached doctoral researchers, who are interested in or even affected by mental health problems, we recognize that this procedure may have created a sampling bias. Further, the nature of the survey questions might have created a demand characteristics bias, potentially leading to elevated levels of reported burden. However, we think that the extent of these biases is comparable to other studies investigating mental health using self-report questionnaires. We also detected a gender response bias: We had more female responders even though we targeted mainly research fields related to neuroscience with a slightly men-dominated gender distribution^16^. BSI scores of our sample were rather low for overall anxiety, but rather high for the subscale phobic anxiety (56%). The latter subscale includes items that might have changed in weighting in pandemic times (e.g., “being in large crowds”), leading to increased scores. We also acknowledge that doctoral researchers’ retrospective assessment of their wellbeing might have led to biased judgements of aspects before the pandemic.

In addition to this limitation, we captured the history of mental illness more broadly (e.g., having contact with psychiatry/psychology or being previously diagnosed with a mental disorder), but did not ask for the specific diagnosis. Thus, our data does not allow for the exploration of the relationship between the mental health indexes and one or another mental disorder in-depth. We recognize that this could be especially important as we observed that a prior diagnosis was the main predictor of several dependent variables. Further, we did not assess the direct impact of the pandemic on the participants (e.g., being infected or having lost relatives or colleagues due to the pandemic). However, we asked participants if they experienced any event within two weeks prior to participation which would render their answers unrepresentative for a longer time perspective and excluded the respective data. We believe that losing a loved one or experiencing high stress due to a COVID-19 infection would have been captured in this response.

On a global scale, our survey focused on German doctoral researchers, with a certain focus on international students within the field of neuroscience, which may be generalizable to countries of similar funding opportunities and research culture. However, limited assumptions should be made about ECRs’ mental health in countries with diverging research conditions (e.g., of the Global South^35^).

### Conclusion

Our findings extend the ongoing debate about the mental health crisis in academia: Doctoral researchers’ self-reports showed increased levels of depression, burnout, and loneliness and further decreased mental wellbeing under the influence of the pandemic. Importantly, self-reported mental health problems were strongly linked to the PhD training, which emphasizes the need to improve academic work culture. Initiatives founded by ECRs have sought to actively reduce harmful work conditions and the stigmatization of mental health problems in academia. However, long-term change also demands top-down solutions. We thus call research institutions to action to create PhD training conditions that target the factors we identified and bring about sustainable systemic changes for academia.

## Materials and methods

### Participants

We sought to target early career researchers pursuing their doctorate at research facilities in Berlin and the greater Berlin area to investigate the status of their mental health and potential COVID-19 related mental health changes via an online survey. The cross-sectional study was approved by the ethics committee of the Faculty of Psychology of the Humboldt-Universität zu Berlin and was conducted in accordance with the Declaration of Helsinki. All participants provided written informed consent. Participants had the option to take part in a raffle to win 25 Euro. In total, 335 doctoral researchers completed the survey. As we aimed to compare the perceived mental health and satisfaction with PhD training of doctoral researchers before and after the start of the COVID-19 pandemic, we excluded participants who did not start their PhD training at least three months prior to the beginning of the pandemic (March 2020 in Germany; n = 75). Additionally, we excluded participants who indicated that they experienced an important life event which could have influenced their mental health significantly in the last two weeks (n = 38). In the remaining sample of 222 participants, the age ranged from 24 to 52 years (*M* = 29.8; *SD* = 3.4). Gender, nationality, years into the PhD training, and type of the PhD program are summarised in Table 3. Type of PhD program refers to structured programs (as often provided by graduate schools) vs. individual doctoral projects. They differ in characteristics, which are potentially important for doctoral researchers’ wellbeing, especially in the pandemic. For example, doctoral researchers in structured programs are often organized in cohorts or years, which facilitates the growth of a professional peer network.

**Table 3:** Participant characteristics.

	Count	Percent
**Gender**		
Woman	143	64.4%
Man	70	31.5%
Other	4	1.8%
Prefer not to answer	5	2.3%
**Nationality**		
German	113	50.9%
Other than German	109	49.1%
**Year into PhD training**		
1st	7	3.2%
2nd	72	32.4%
3rd	46	20.7%
4th	58	26.1%
5th	23	10.4%
6th	12	5.4%
> 6th	4	1.8%
**Type of PhD program**		
Individual	101	45.5%
Structured	106	47.8%
Other	8	3.6%
Do not know	7	3.2%

### Procedure

Data were collected cross-sectionally between January and February 2021 with an online survey administered via the SoSci Survey platform (www.soscisurvey.de) and further processed in R ver. 4.0.2. The R code including data, analysis code and a HTML file with all procedures rendered in accessible form, are available at an OSF repository (https://osf.io/q5w4g/). The survey consisted of standardized measures of mental health and self-developed questions regarding changes in satisfaction before and during the pandemic in relation to PhD-specific aspects.

#### Standardized measures of mental health

We included three standardized questionnaires to assess participants’ mental health state. For all these questionnaires, higher scores relate to greater significance of the measured mental health problem. We used the Brief Symptom Inventory (BSI^54^), which has 53 items to assess nine mental health symptom dimensions: somatization, obsession-compulsion, interpersonal sensitivity, depression, anxiety, hostility, phobic anxiety, paranoid ideation, and psychoticism. Participants were asked to choose how much a described problem bothered them in the last week (e.g., “Nervousness or shakiness inside”, scale: “Not at all”, “A little bit”, “Moderately”, ”Quite a bit”, and “Extremely”). For each dimension, raw scores were transformed into t-scores to relate the results to the general population. In reference to the BSI manual^54^, we considered t-scores equal to or larger than1 SD (t-score = 60) as clinically relevant.

The Copenhagen Burnout Inventory (CBI^15^) was used to measure personal and work-related burnout (the third CBI scale, namely client-related burnout, was omitted in our survey as the work of doctoral researchers is rarely concerned with clients). In 13 questions, participants were asked how often they experienced described problems in the last weeks (e.g., “How often do you feel tired?”, scale: “Never/Almost never”, ”Seldom”, ”Sometimes”, ”Often”, “Always”) or how intense these problematic experiences were (“To a very low degree”, ”To a low degree”, ”Somewhat”, ”To a high degree”, ”To a very high degree”). For scoring, each answer is assigned a numerical value from 0 for “Never/almost never” or “To a very low degree” to 100 for “Always” or “To a very high degree”. The score is calculated as the mean of all items.

Further, we used the De Jong Gierveld Loneliness Scale (11-item version^55^) to measure social and emotional loneliness. Participants were asked to indicate how often they experienced different situations (e.g., “There is always someone I can talk to about my day-to-day problems”, scale: “None of the time”, “Rarely”, “Some of the time”, “Often”, “All the time”). Maximum total score is 11 (one per item), which combines six items from the emotional subscale and five items from the social subscale.

#### Self-developed questions on mental health and satisfaction with PhD training under consideration of the pandemic

In order to assess participants’ perceived mental health and satisfaction in relation to their PhD training, we created questions specifically targeting these topics considering the context of the pandemic situation. We based wording and content of the questions on other surveys targeting ECRs’ mental health, such as the survey of the N^2^ network^16^ and the annual Nature survey^3^. We subsequently adapted the questions to capture the influence of the pandemic.

Besides demographic questions and PhD specifics (e.g., years into PhD training), we collected information on existing and past clinical mental health diagnosis, expectations regarding the PhD training before its start, potential support structures, and changes in satisfaction with PhD-specific aspects (e.g., supervision) caused by the pandemic. We thus asked our participants to evaluate these aspects retrospectively for the times before the pandemic as well as the current in-pandemic situation. The results reported here include a subset of all questions administered in the survey; the full list can be found in the OSF repository (https://osf.io/q5w4g/).

### Statistical analyses

To conceive the actual mental health state of our participants, we used descriptive statistics for the standardized questionnaires. Therefore, we computed the mean and standard deviation of the BSI, CBI, and De Jong Gierveld Loneliness Scale scores to compare these values with existing literature. For the self-developed questions on mental health of doctoral researchers and ratings of satisfaction with PhD training, we calculated percentages per response category. We then performed two exploratory statistical analyses: The first analysis aimed to investigate whether there was a change in retrospective pre-pandemic and current in-pandemic ratings of PhD-related satisfaction aspects and items contributing to mental health issues. We performed two-tailed dependent t-tests for all related items. The second analysis aimed to investigate potential predictors of three mental health aspects: depression, anxiety, and burnout. We chose these aspects because they are most frequently considered in the context of the mental health of ECRs^2–4^. To this goal, we built three multiple regression models with the BSI depression t-scores, the BSI anxiety t-scores, and the CBI work-related burnout scores as dependent variables. To control for mental health issues potentially unrelated to the PhD, we included prior clinical diagnosis of a mental disorder (pre- or since the beginning of the PhD) as a control variable in all models. We expected that the three mental health aspects would be related to years into the PhD training, overall satisfaction with the PhD training, being a member of a graduate program, and gender, but we were also interested in the contribution of other factors. Therefore, we entered all suitable survey questions (see column “Predictor” within the HTML file on the OSF page) as potential predictors in the models, and conducted a stepwise selection of the best model with the bidirectional step() function (stats package). This procedure allows for iterative adding and removing predictors based on the model’s fit estimated with the Akaike Information Criterion (AIC). The predictors in the selected models are chosen based on their statistical significance for explaining variance. Due to few data points in the following categories, we excluded participants who (1) did not identify as a woman or a man (n = 9), (2), did not indicate being part of a structured or individual PhD program (n = 14), and (3) chose not to provide information about whether they were diagnosed with a mental disorder (n = 1). This procedure resulted in a sample size of 198 participants for this exploratory analysis. Responses to the question “How long have you been living in Germany?” were binned into three categories: 0–3 years, 4 + years, and native. Due to the design of the survey (participants could not proceed without giving a response for each item concerned for analysis), there was no missing data.

It should be noted that stepwise regression is a discouraged method for hypothesis testing and should be interpreted with caution in data exploration. The reason for this is that it may produce narrow confidence intervals, high t statistics and low p values^56^. However, when treated with caution, it could generate valuable insights about the relationships in the dataset and produce ideas for future hypothesis testing studies. Further, because stepwise regression is less effective with more explanatory variables^56^, we did not include interaction terms in our exploratory models. Finally, because the *p* values in stepwise regression are not accurate and should not be interpreted as they would in a hypothesis-testing, theory-driven analysis, we do not correct for multiple comparisons, as there is no straightforward method to do so and no apparent benefit for the interpretability of the results. With all this in mind, in the current study, we do not encourage other researchers to use the reported statistics as true effect sizes in the population, but rather to consider this procedure as an exploratory tool for building insights about our data and generating future hypotheses in theory-driven studies.

The stepwise selection procedure confirmed that prior diagnosis explained significant portions of variance in all models. Hence, other predictors explained additional variance in the data suggesting that PhD-related aspects may have a somewhat additive (to clinical diagnoses) influence on mental health problems. We also explored models without prior diagnosis as a predictor, which showed a similar pattern of results as the full models presented below (see point 6.2 in the HTML file in the repository). The selected models were checked for regression assumptions (normality, linearity, multicollinearity (with Variance Inflation Factors), homoscedasticity), which were met for all models (details for each model are reported in the HTML file). The significance level for all the tests was set to 0.05. However, it should be noted that these analyses are data-driven and thus any statistical significance should be considered with care. To estimate the main effects in the analyses (across levels of multiple categorical predictors), we conducted a type-II analysis of variance using the model parameters.

### Ethics approval

The study protocol was reviewed and approved by the ethics committee of the Department of Psychology at Humboldt-Universität zu Berlin. The study was conducted in accordance with the Declaration of Helsinki.

## Data Availability

Data and code that support the findings of this study are openly available at the OSF repository: https://osf.io/q5w4g/.
